# Opioid use trends in Spain: the case of the island of La Gomera (2016–2019)

**DOI:** 10.1007/s00210-021-02193-0

**Published:** 2021-12-09

**Authors:** Alexis Oliva, Néstor Armas, Sandra Dévora, Susana Abdala

**Affiliations:** 1 Departamento de Ingeniería Química Y Tecnología Farmacéutica, Facultad de Farmacia, Universidad de La Laguna, Tenerife, Spain; 2 Departamento de Medicina Física Y Farmacología, Facultad de Farmacia, Universidad de La Laguna, Tenerife, Spain

**Keywords:** Opioid prescribing, Wholesaler, Defined daily dose, Socioeconomic status, Rural, Spain

## Abstract

This study is an evaluation of prescription opioid use on the island of La Gomera, a mainly rural area, during the period 2016–2019 at various levels. Data were extracted from the wholesalers who supply the community pharmacies at the population level. Prescription opioid use was measured as defined daily doses per 1,000 inhabitants/day (DID) and by the number of units sold per 1,000 inhabitants and year (units sold). This provided an island total of La Gomera’s overall prescription of opioids and its rate of change, as well as differences in prescribing at the municipal and health area level. Tramadol with acetaminophen and tramadol in monotherapy were the most consumed by “units sold” parameter, which accounted for 69.48% and 18.59% of the total. The situation was similar for DID, although with lower percentages, but a significant increase was observed in the use of fentanyl and buprenorphine, around 15% in each case. The balance between the uses of weak or strong opioids was different in La Gomera compared to that of Spain as a whole. In Spain, almost 70% of the prescriptions were for weak opioids compared to 58.67% in La Gomera. Fentanyl was the most used strong opioid (16.10%) followed by tapentadol and buprenorphine, around 5% each, whereas in La Gomera, buprenorphine was the most consumed (15.75%) followed by fentanyl (14.87%) and tapentadol (5.82%). These differences in prescription opioid use are most likely explained by prescriber characteristics, whereas the population age, socioeconomic status, or living in rural/urban area are not decisive determinants.

## Introduction

A drug utilization study (DUS) includes the analysis of the commercialization, distribution, prescription, and use of medicines in the society, with special attention paid to the results of medical, social, and economic consequences (WHO 2020). A DUS can be useful to know the pattern and use of different medicines and their evolution over time, and it can compare the data from one region to another and thus contribute to a more responsible use of them (Laporte and Tognoni 1993). The DUS here used the prescription data from a nationwide drug data bank as the main source of information (Hamunen et al. 2008; Jarlbaek 2019). For example, each country’s prescription database can be accessed on the Internet to obtain more detailed information on the annual number of prescribed opioids (Jarlbaek 2019). Knowledge of the number of prescribed opioids for a determined geographic area (such as a municipality or an island) are not available in such a database.

The treatment of chronic pain comprises both pharmacological and non-pharmacological strategies (Merskey and Bogduk 1994; Collins et al. 1997; Dale et al. 2005). Among the former are non-opioid pain relievers, opioids, and adjuvants (used to prevent or treat side effects of pain relievers or to enhance the pain relief itself).

Opioid drugs are a group of drugs characterized by having a selective affinity for central and peripheral opioid receptors, inhibiting the transmission of the nociceptive entry and the perception of pain (Dworkin 2002; McCleane 2004; Dowell et al. 2016). They are widely accepted for the treatment of severe acute pain and chronic moderate to severe pain that does not respond to other medications. The use of these drugs is associated with the development of physical dependence and addiction, which has been a major health problem in developed countries due to the potential risk of abuse of these substances (Boudreau et al. 2009; Zhu et al. 2019; Bosetti et al. 2019). The use of prescription opioids has increased in the last decade in many developed countries (Hamunen et al. 2009; Hastie et al. 2014; Hider-Mlynarz et al. 2018; Alexander et al. 2018). For instance, the abuse of prescription opioids in the treatment of pain in the USA and the devastating consequences of overdose are well known (INCB 2017; Hamunen et al. 2009). The prescription pattern of opioid use also varies considerably between countries, especially, in European countries, not only regarding total prescriptions but also the preference for different opioids (Bosetti et al. 2019). For example, fentanyl is the most frequently consumed opioid analgesic in many countries (García del Pozo et al. 2008; Hamunen et al. 2009; Hider-Mlynarz et al. 2018), although Mordecai et al. (2018) indicated that fentanyl was, by far, the least prescribed drug in primary care in England during 2010–2014. However, previous studies focused on describing how opioid prescription rates vary across country, region or province-municipal-level, or between rural and urban areas (Shoff et al. 2021). Other authors have examined demographic and socioeconomic factors related to prescription opioid use in different countries (Bosetti et al. 2019; Jarlbaek 2019; Pear et al. 2019; Böckerman et al. 2020). The main conclusion was that prescription opioid use appears to be more common among low socioeconomic status people. Other studies have shown that there are considerable differences in opioid-related measurements between rural and urban areas. The rural areas were found to have higher rates of opioid prescription, higher rates of high-dose opioid prescription, or fewer resources for inpatient and outpatient opioid treatment, etc., in comparison to urban areas (Keyes et al. 2014; Sears et al. 2020; Shoff et al. 2021). Serdarevic et al. (2017) suggest that older women, especially, those living alone, have higher rates of prescription opioid use.

Although prescription opioid use has been studied in various countries in the last 2 decades (García del Pozo et al. 2008; Hamunen et al. 2009; Hastie et al. 2014; Hider-Mlynarz et al. 2018; Alexander et al. 2018; Zhu et al.^2019^; Bosetti et al. 2019), few studies in Europe have focused on local zones such as islands, municipalities, or health areas to establish a prescription pattern of opioid use. For example, Mordecai et al. (2018) examined patterns of regional variation of opioid prescription in primary care in England during the period 2010–2014.

The aim of this work is firstly to evaluate the prescription opioid use on the island of La Gomera, Canary Islands, Spain, as example of an area with a mainly rural population. In order to do this, a drug utilization study based on the Anatomical Therapeutic Chemical classification (ATC) and defined daily dose (DDD) methodology was used (WHO 2018). Secondly, to detect any deviations in the prescription opioid use rates at various levels (nationwide and local) as well as to analyze the possible changes during the study period. Thirdly, the demographic and socioeconomic factors which were possibly related to prescription opioid use were examined in a population who live in a mainly rural area. To accomplish this, the raw data obtained from the wholesalers who supply the community pharmacies at the population level were used as the database, which is novel.

## Material and methods

The study of prescription opioid use at the inpatient level was conducted using the ATC/DDD methodology, which is internationally accepted for measuring drug utilization within and across populations. The different opioid types were identified in the database by their ATC code. Opioids indicated for analgesia all start with N02A. A few other opioids are also relevant as analgesics, such as methadone and codeine. These drugs are not routinely included in the usual opioid use statistics, because they are not only indicted for analgesia (Jarlbaek 2019). The subgroups of the ATC classification studied here are N02AA (natural opium alkaloids), N02AB (phenylpiperidine derivatives), N02AE (oripavine derivatives), N02AJ (opioids combined with other pain relievers, except codeine with combinations), and N02AX (other opioids).

The present study used the raw data obtained from wholesalers who supply the community pharmacies at the population level on the island of La Gomera during the period 2016–2019. The data were provided by the Pharmaceutical Cooperatives of the Canary Islands. Only data on opioids sold in the pharmacies were included because this use must be linked for legal reasons to the prescription in health records.

The data provided were the number of units sold of the different pharmaceutical preparations according to the ATC classification, date of sale, national code, and pharmaceutical preparation name (active pharmaceutical ingredient (API), dose, strength, and units). The post code (ZIP) was also included, given that this data provides information about the municipality where the pharmacy is located, but not its identity, thus maintaining its anonymity in accordance with current data protection law in Spain (2018).

The prescription opioid use was measured in defined daily doses (DDDs) or in the number of medication packages (boxes or units sold). The former indicator provides a fixed unit of measurement that accounts for the differences in package size and strength, making comparisons possible between population groups or geographical areas. In order to do this, prescription opioid use was expressed as DDDs/1,000 inhabitants/day (DID), where the number of DDDs was the total amount of the API consumed in a certain time period (in this case, day) divided by the DDD.

$$DID=\frac{DDDs\times 1000}{populations \times 365 days}$$

where *DDD* represents the total number of *DDD*s, which are set by the WHO and published on the website of the *WHO Collaborating Center for Drug Statistics Methodology* (WHO 2018).where UD is the unit dose in mg, N is the number of units per package, and DDD is the defined daily dose.

The second indicator used was the units sold, expressed as the number of packages per 1,000 inhabitants and year (Packs/1,000/year).

$$units sold=\frac{number of packages\times 1000}{number of inhabitants}$$

Population data were downloaded from a publicly accessible demographic database and are shown in Table 1 (National Statistics Institute in Spain 2020). The database and dynamic tables of the Microsoft Excel® program were used for the data processing.

**Table 1:** Demographic data and administrative distribution of the island of La Gomera (Istac, 2020). The population density is expressed as number of inhabitants per km^2^ in 2020

Municipality	2016	2017	2018	2019	Health area	Population density
Agulo	1,074	1,066	1,067	1,096	G02	43.2
Alajeró	1,971	1,983	2,006	2,017	G06	40.8
Hermigua	1,924	1,808	1,805	1,822	G02	45.9
San Sebastián	8,707	8,760	8,945	9,093	G01	79.4
Valle Gran Rey	4,285	4,371	4,484	4,564	G04	141.0
Vallehermoso	2,979	2,988	2,829	2,901	G03, G05	26.5
Total inhabitants	20,940	20,976	21,136	21,493	6	

## Results

### Data analysis at the island level

#### Units sold indicator

**Fig. 1:**
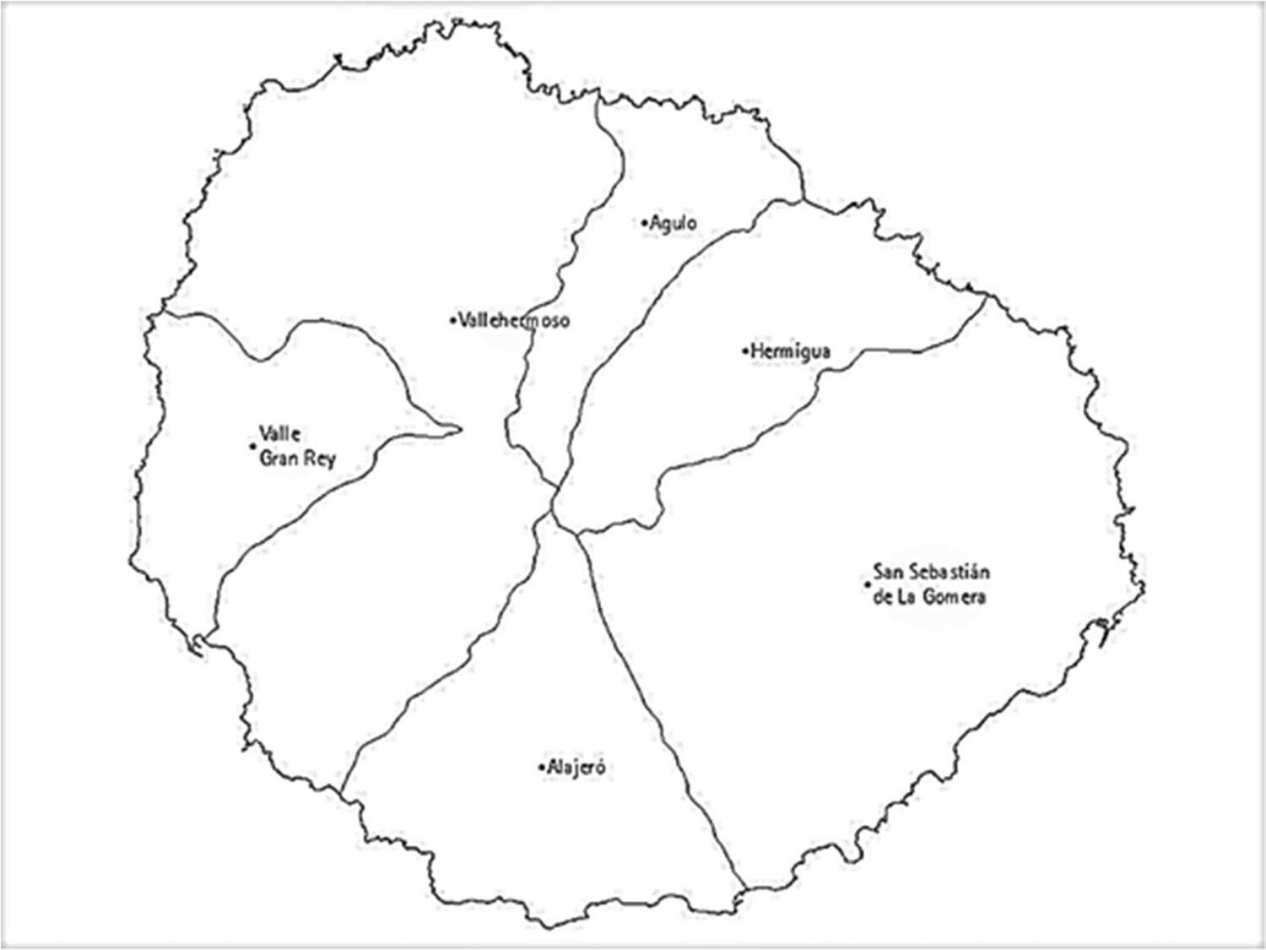
La Gomera Island with its six municipalities

Figure 1 shows the map of the island of La Gomera, with its six municipalities and health areas, with its capital being San Sebastián de La Gomera (Grafcan 2020).

**Fig. 2:**
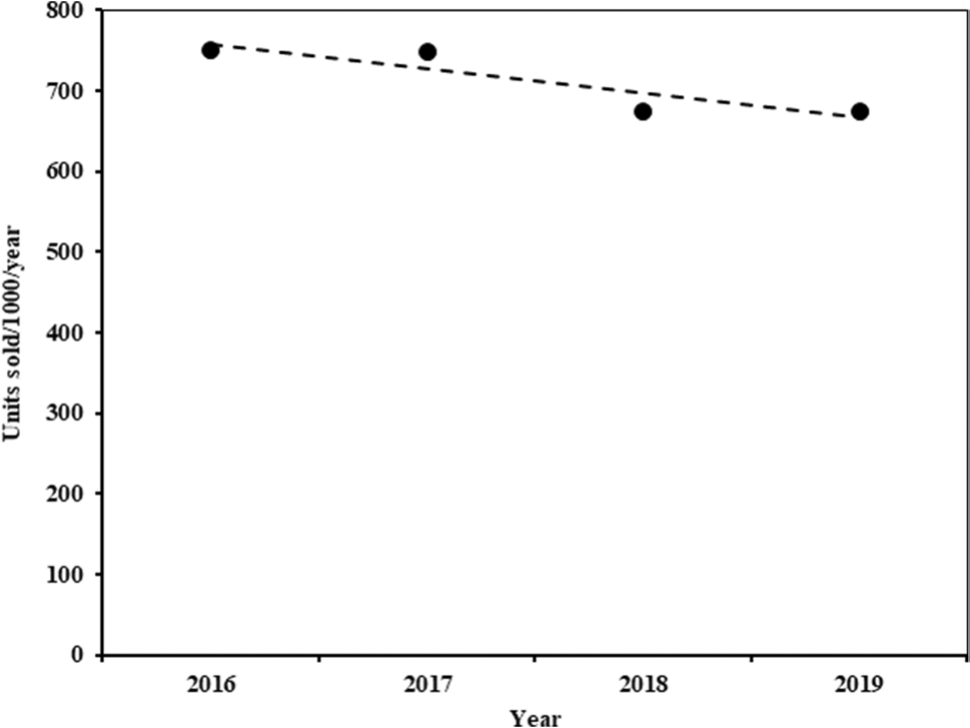
Variation of the number of packs per 1,000 inhabitants and year in the island of La Gomera over a 4-year period (2016–2019). A reduction of 2.52% (on average) was observed in the number of units sold, whereas the population increased by 2.64% in the same period

Figure 2 shows the variation of the sold unit’s indicator during the study period. The data analysis shows a negative correlation (*r* = 0.90) between both variables, whereas the population at island level increased in the same period. This result implies a decreased in the prescription opioids. Table 2 shows the number of units sold (expressed as a percentage) by therapeutic subgroup/API and year. The tramadol-acetaminophen combination was the number one option in the tablet pharmaceutical form and presentation at low-dose and low-unit per packages (37.5 mg/325 mg; 20 units) accounting for 74.13% of the total versus 13.25% for 60 unit presentation in 2016. However, this tendency changed at the end of the mentioned period due to the increase of the sales of the generic equivalent forms from 20 to 25%. Prescription for presentations with high-dose and high-unit per packages (75 mg/650 mg; 60 units) was 12.6% of the total, but this tendency decreased to 7.13% in 2019. The tramadol-dexketoprofen combination is noteworthy for its increase in 2019; its prescription almost doubled compared to 2017, the year of its introduction in the market, from 5.62% in 2017 to 10.29% in 2019.

**Table 2:** Variation of the two indicators (expressed as a percentage of the observed mean value during the analyzed period) by subgroup and API in La Gomera and in Spain. Data from Spain were extracted from the report on the “Use of opioid drugs in Spain during the 2010–2019 period” published by The Spanish Agency for Medicines and Medical Devices in 2019 (AEMPS 2020)

	La Gomera		Spain
Subgroup /API	% units sold	% DID	% DID
N02AA01-Morphine	2.22	1.83	1.58
N02AA03-Hydromorphone	0.09	0.12	0.29
N02AA05-Oxycodone	0.40	0.24	0.67
N02AA55-Oxycodone_Naloxone	1.58	2.71	3.49
N02AB02-Pethidine	0.02	< 0.001	0.02
N02AB03-Fentanyl	6.29	14.87	16.10
N02AE01-Buprenorphine	1.33	15.75	4.62
N02AJ13_Tramadol_Acetaminophen	63.35	34.73	49.43
N02AJ14_Tramadol_Dexketoprofen	6.12	3.98	1.27
N02AX02-Tramadol	13.80	19.96	17.58
N02AX06-Tapentadol	4.79	5.82	4.95

On the other hand, the prescription buprenorphine use is characterized by high-dose and long-acting preparations (transdermal patches, 70 µg/h, 5 units), and low-doses and short-acting formulations (tablets, 0.2 mg, 20 units) accounting for 25% and 35% of the NO2AE subgroup total prescriptions, respectively. At present, the prescription of both pharmaceutical formats is similar.

A change in tendency was also observed in prescription morphine use from 20.7 units sold in 2016 to 13.9 in 2019; this represents a reduction of 29.6%. The high-dose formulation (10 mg/12 tablets) was the most consumed. Regarding oxycodone, a marked reduction in prescription was observed, with a decrease of 96% of the total of units sold in 2019 in comparison to the data observed in 2016. However, oxycodone-naloxone prescription was stable, around 1.58% on average, with the high dose and low units per package (10 mg/5 mg and 20 mg/10 mg with 28 tablets) presentation having the greatest demand. The presence of hydromorphone and pethidine were negligible.

In the case of the fentanyl, the most frequently prescribed pharmaceutical forms throughout the study period were transmucosal lozenges (400 mg/30 units) and patches (12 mg and 25 mg/5 units). The presence of fentanyl in the form of nasal spray was low, 1.31% in 2019 compared to 8.5% in 2017.

Tramadol is prescribed as long-acting and low-dose (50 mg) tablets, and accounted for around 15% of the total in the first 2 years, with its demand doubling in 2019. It should be mentioned that the oral solution pharmaceutical form had a demand close to 13.3% of the total in the last 2 years, after peaking in 2017 with 22.6% of the total. A similar situation was observed for tapentadol derivatives.

#### DID indicator

**Fig. 3:**
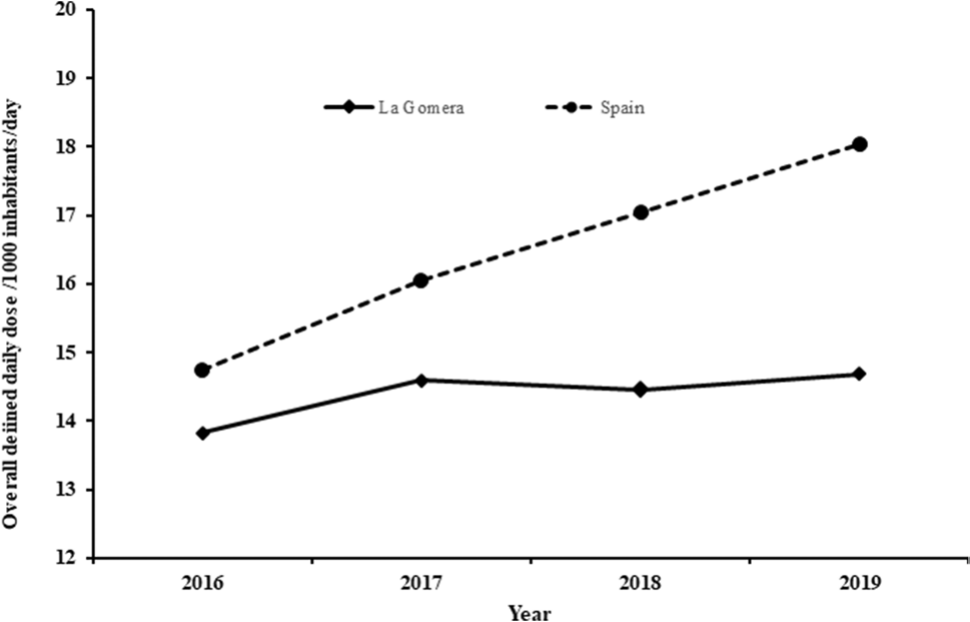
Variation of the overall DID in La Gomera and Spain as whole during the study period. In Spain, the overall mean annual DID rate increase was 0.07% with a negative tendency, whereas in La Gomera, this rate has been stable, especially, over the last 3 years

**Fig. 4:**
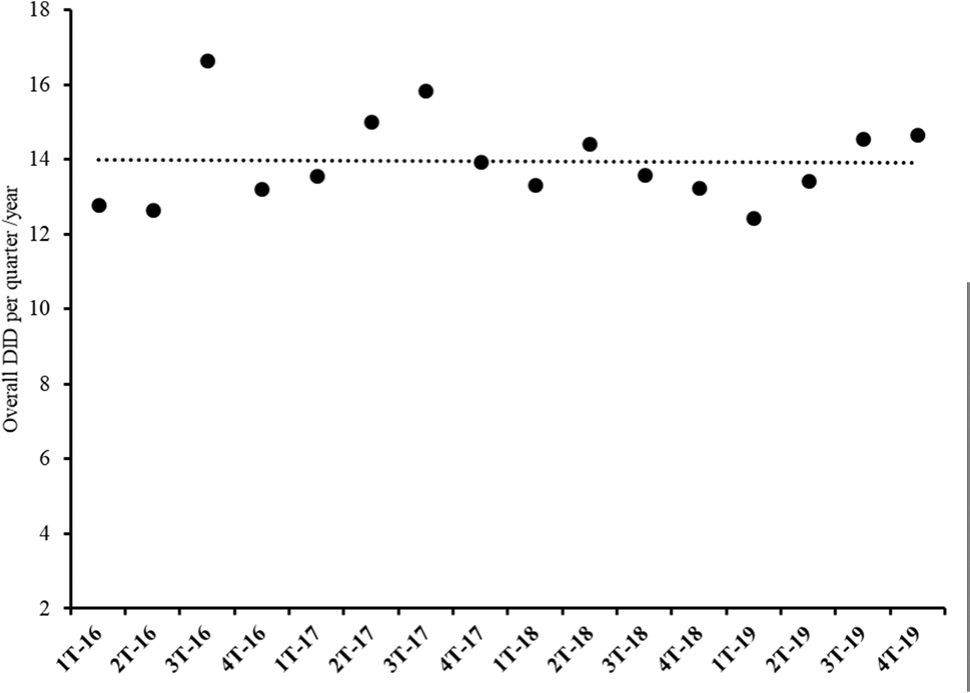
Variation of the overall DID per quarter and year on the island of La Gomera during the study period. This variation can be considered constant, with a mean value of 14 DID with 95% confidence intervals (13.33, 14.58). All individual values are within the $\overline{X }\pm 3s$ d range

Figure 3 shows the variation in overall DID in La Gomera. This varied from 13.83 DID in 2016 to 14.69 DID in 2019, which is an increase of 6.2%, although it remained fairly stable over the last 3 years. However, the population only increased by 2.64% (see Table 1) in the same period. Figure 4 shows the evolution of the DID per quarter and year. The prescription per quarter presents fluctuations over the four-year period. The peak was reached in the third quarter of 2016, followed by a sharp decrease, and later DID increased in the following three quarters again. Nevertheless, from that time until the beginning of 2019, there was a continuous and slow decrease in DID, except in the second quarter of 2018 where a slight increase was observed, later returning to values close to those of 2016. The reason for this change in the tendency could be related with the introduction of the co-payment of medicines by pensioners; 21.4% of the population of La Gomera is over 65 years old, which is more than 5 percentage points above the average in the Canary Islands, 15.6% (Istac 2020). In addition, the slight rebound in prescriptions observed during 2019 could be related to the withdrawal of the co-payment mentioned above.

During this period, the most prescribed opioid was tramadol-acetaminophen followed tramadol in monotherapy, derivatives of the buprenorphine, and the phenylpiperidine. Tapentadol and tramadol in combination with dexketoprofen were in fifth and sixth position, respectively (see Table 2). Among the natural opium derivatives, the prescription oxycodone-naloxone use remained steady throughout the period, 2.71% on average, whereas there were fluctuations of around 2% in the prescription of morphine. The prescription of hydromorphone and oxycodone was less than 0.4% of the total.

### Data analysis at the municipal and health area level

Table 3 shows the DID obtained per municipality and year during the 2016–2019 period. The data shows the high variability among all the municipalities during the study period. Alajeró and Hermigua, two of the smallest municipalities, with approximately 2,000 inhabitants each, show DID values above the annual DID at the island level (Table 3). In contrast, Valle Gran Rey, with half the population of the capital, and Vallehermoso, with just under 3,000 inhabitants, have a lower DID. However, the prescription pattern was similar among the municipalities and the island as a whole, where the combination of tramadol-acetaminophen was the number one choice in all of them, followed by tramadol, fentanyl, and tapentadol, whereas the natural opium derivatives occupied the last position in the ranking. At the municipal level, the data indicates that, on an average, San Sebastian and Valle Gran Rey, with a higher population density living in an urban area, had lower prescription opioid use rates (Table 3). In addition, both of the latter municipalities have the smallest proportion of elderly people (17.4 and 21.0%), and their economy is based on tourism and related activities (≈ 80%). Most of the remaining residents in the other municipalities live in rural areas with a disperse population in small villages dedicated to agriculture (60–70%) and where the most elderly people live (Agulo and Vallehermoso have values above 30%). At first, the prescription rate should increase with the rise in the older people rate. The results show that there is not significance correlation between the prescription opioid use rate, expressed as DID, and the older people rate at municipal level (*r* = 0.351, *p* < 0.01).

**Table 3:** Variation of DID by municipality and year in La Gomera during the period 2016–2019

Municipality	2016	2017	2018	2019
Agulo	12.76	13.95	16.56	14.67
Alajeró	18.28	18.15	17.55	18.75
Hermigua	15.62	15.50	19.97	20.61
San Sebastián	12.55	12.84	14.78	15.15
Valle Gran Rey	12.00	10.40	10.02	10.86
Vallehermoso	10.83	12.51	7.87	5.90
Island DID	13.83	14.59	14.45	14.69

In the case of “units sold,” the data analysis shows that the capital accounts for 45% of the sales but has 42% of the island’s total population. Alajeró together with Hermigua make up 14% of the island’s population with a similar percentage of sales, around 9% each. Valle Gran Rey has a slightly higher consumption in comparison with the total population, 20.9% vs 20.4%. An exceptional case is Vallehermoso where there was a significant decrease in the units sold, from 113.4 units in 2016 to 43.9 in 2019; this is a decrease of 65.5%, while the population has also decreased, but at a lesser rate, only 2.62%. The reason for this change in the tendency could be related to sociodemographic factors. Vallehermoso is the largest municipality in terms of surface area and has a low population density since part of its territory is national park and forest or other uninhabited areas. Furthermore, the only community pharmacy is located in the north of the municipality, whereas the population who live in the south (minority) get their medication in a pharmacy in another municipality.

Despite the differences in population and units sold between municipalities, differences in DID persist among the health areas, similar to those individually observed for the municipalities. The G01 health area (see Table 1) had a 15.15 DID in 2019, while the G04 health area had a 10.86 DID. Both areas have a similar “units sold/population” ratio, and therefore, their DID should be similar. However, this relationship is not enough to explain this difference. The explanation could be found in the increase in prescriptions of those pharmaceutical preparations and presentations with a higher DDD. For example, in 2019, the contribution to the total DID from formulations containing tramadol or in combination with acetaminophen and dexketoprofen was similar in both areas (8.08 vs 8.63). The number of units sold of the abovementioned formulations accounted for 83.04% in the G01 area compared to 91.25% in G04 (582.1 vs 523.7). Nonetheless, the contribution of the formulations containing buprenorphine and fentanyl was 5.78 DID versus 1.54 DHD in the G04 area. These two APIs have a low DDD (1.2 mg) in comparison with tramadol-acetaminophen (3 g). In terms of units sold, this was 10.20% of the total for the G01 area, while this was 4.43%, (71.5 vs 25.2 units sold) in G04. This was repeatedly observed throughout the analyzed period with minor variations.

## Discussion

The data obtained with the two indicators used in the DUS seem to show different results and conclusions, in such a way that both the classification by importance of consumption (expressed as units sold) and the percentage of each subgroup based on DID varied considerably (Table 2). This can be explained by the fact that the two parameters used have different purposes. The use of the “units sold” indicator provides information on the guidelines for the use of a specific therapeutic subgroup within a defined geographical area, in the case here, the island of La Gomera and its municipalities. The ranking of different opioid categories was fairly stable in all the municipalities, although the percentages varied slightly. This difference in pattern is due to variations in the number of units sold that do not necessarily reflect a real variation in consumption.

At the island level, the tramadol-acetaminophen combination accounts for 63.35% of the units sold total, while in DID, it is 34.73% of the same total. Tramadol ranks second in both the number of units sold and in its contribution to DID. Natural opium derivatives represent 4.30% of total prescriptions (and fourth place in order of prescription), with 5.19% being in DID, thus ranking last. Buprenorphine is only 1.33% of the units sold total, lower than morphine and oxycodone-naloxone, but it ranks third in its contribution to DID with 15.75%. In the case of fentanyl, it is 6.29% of the number of units sold, in comparison with 14.87% in DID, where it is in the fourth position in DID.

The Spanish Agency for Medicines and Medical Devices (AEMPS) published in June 2019 its report on the “Use of opioid drugs in Spain during the 2010–2019 period” (AEMPS 2020). According to the report, opioid consumption in Spain increased from 10.03 DID in 2010 to 19.83 DID in 2019, doubling the value in a decade, although in the last 4 years (2016 to 2019) the increase was lower, 22.3%. A study of opioid consumption in three Scandinavian countries during 2006–2014 in terms of DDDs showed that Denmark presented DID values close to 20, whereas Norway had the lowest consumption (≈17 DID), although a small reduction was observed for Sweden during the said period.

The present study excluded codeine combined with other analgesics since these drugs may be used in other pathologies (Jarlbaek 2019). Therefore, and for comparative purposes, the data of the nationwide report were re-analyzed to take this fact into account (Fig. 3). The recalculated DID varied from 14.75 in 2016 to 18.04 in 2019, values that are above the overall DID at an island level (see Fig. 3). During this period, the observed increase in Spain was three and half times higher than in La Gomera. The nationwide data seems to suggest a greater use of prescription opioids, but the increase in population does not justify this observation. Although the data expressed in DID provides more accurate information on prescription opioid use than simply using the number of units sold, the unexpected findings suggest that the problem should be analyzed from another point of view, for example, by analyzing data on strong or weak opioids separately. Opioids are classified in two groups—weak (tramadol in monotherapy and combinations) and strong opioids (the remaining ones). This type of classification was used by Svendsen et al. (2011), Jones et al. (2020), and by Jarlbaek (2019) in their studies on opioid consumption.

At the island level, the data analysis based on this classification shows that weak opioids are the most consumed, especially, those presentations with lower-dose and lower number of units per package; however, with respect to strong opioids, the low dose and low units per package is not always the most consumed form. In the case of fentanyl, the transmucosal formulation with high-doses (400 μg dose, 30 lozenges) and the patches, especially, with a low dose (12 µg/h and 25 μg/h doses) and five units are the most frequently consumed. Both formulations are indicated for the management of breakthrough pain in cancer patients. A similar situation was observed with buprenorphine, with the long-acting preparations being the most prescribed preparations, accounting for 55% of the total. In the case of natural opium derivatives, the high-dose and short-acting preparations were the first option.

The consumption of weak opioids (as DID) in Spain represents 68.28% of the total; tramadol in combination with acetaminophen was the most consumed, followed tramadol in monotherapy, indicated for the treatment of moderate to severe intensity pain, especially in older people since it is prescribed in preference to non-steroidal anti-inflammatory drugs because of concerns over complications (Harirforoosh et al. 2013). This tendency in the prescription of tramadol is not an isolated event. The use of tramadol has increased worldwide over the last decades. For example, in Denmark, its use doubled from 2011 to 2013 (Muller et al. 2019), although a decline in prescription tramadol use was observed from 2014 to 2019 (Sorensen et al. 2021). Mordecai et al. (2018) mention several reasons for the increase in tramadol prescription and its consequences on public health in the UK.

In contrast, strong opioids account for 31.72% of the total, with fentanyl being the most prescribed, followed by tapentadol, buprenorphine, and oxycodone combined with naloxone and morphine. All of the abovementioned are prescribed for the treatment of pain with precise indications. Fentanyl is indicated for cancer patients with breakthrough pain and buprenorphine is indicated for the treatment of moderate to severe cancer pain and severe pain that does not respond to non-opioid analgesics (Dowell et al. 2016). A recent study reports a relevant use of fentanyl in non-cancer patients (González-Bermejo et al. 2021). The easy administration of transdermal and transmucosal formulations could explain the increase in fentanyl prescription, particularly in western and northern European countries as well as in Spain (Bosetti et al. 2019; González-Bermejo et al. 2021). Oxycodone-naloxone is indicated for the treatment of severe pain that can only be adequately treated with opioid analgesics, which explains its low prescription rate.

The comparative analysis between Spain and the island of La Gomera shows a noticeable variation in the prescription of weak and strong opioids. In La Gomera, weak opioids (as DID) represent 58.67% of the total tramadol with acetaminophen being the most frequently prescribed, approximately 10 percentage points lower than in Spain as a whole, whereas the percentage of tramadol indicated in monotherapy is slightly higher in La Gomera (19.96 vs 17.58). The remainder corresponds to tramadol-dexketoprofen. However, the percentage of prescribed strong opioids is 41.33%, higher than that observed nationwide, which is 31.72%. In this case, fentanyl and buprenorphine are the most consumed strong opioids, each accounting for an average of approximately 15% of the total. Among the natural opium derivatives are morphine (1.83%) and oxycodone-naloxone (2.71%), although their percentages vary slightly, but their net contribution is the same, around 5%. The consumption of oxycodone, hydromorphone, and pethidine represents barely 0.36% of the total, while the respective values in Spain as a whole were close to 1%. In this regard, the adverse effect profile of morphine has limited its use and oxycodone is often used as an alternative drug for affected patients (Mordecai et al. 2018).

This difference in prescription pattern could be related with sociodemographic characteristics and health-related factors, among others. Approximately, 60% of the population in La Gomera is concentrated in the capital and Valle Gran Rey municipality with a high socioeconomic status with easy access to all essential services, whereas the population in the smaller municipalities is highly disperse, with low socioeconomic status, a lower population density (on average, 40%), characterized by small villages, far away from essential services and with a high proportion of elderly people. The proportion of 65 + aged people is 21.4% of the population (on average) of La Gomera, second only to the island of El Hierro with 22.3% (Istac 2020). All municipalities have primary care services and, at least, one community pharmacy. Approximately 90% of the patients get their medication from a pharmacy close to where they live. However, the residents of far way municipalities such as Agulo and Vallehermoso can buy their medication in a pharmacy located at San Sebastian when they visit the specialist physician beginning their treatment (≈10–20%). Thus, the variations in overall DID could be overestimated at municipal level. The potential bias is difficult to quantitate since the prescription is associated pharmacy postal code (ZIP) and not township where the patients live.

Different researchers report that the amount of prescription opioids has increased with the rise in the percentage of the urban population in a region, as access to medical care services is better (McDonald et al. 2012; García et al. 2019). In contrast, older people may have unique requirements for prescription opioids due to aging. Older people report more pain conditions, especially musculoskeletal pain, and they are more likely to be prescribed with opioids than the younger population (Zin et al. 2014). The age structure of the population, overall health conditions, and socioeconomic status are the main factors influencing the extent to which they need opioids to treat their illness. On the other hand, a prescriber’s specialty may be directly related to the medical conditions that may be best treated with opioids. The clinicians can find themselves facing a dilemma; they should prescribe the less powerful opioids but with a few adverse effects. However, poorly controlled pain is a public health issue and the personal, familiar, and societal costs cannot be measured, especially, in older age groups. All these factors should be taken into account in the therapeutic strategy (O’Brien et al. 2017).

There are studies that have demonstrated that prescription opioid use rates are much higher in more socioeconomically deprived areas (Mordecai et al. 2018); others suggest that older women, especially those living alone, have higher rates of prescription opioid use (Serdarevic et al. 2017). Another study reported that rural areas are subject to higher rates of opioid prescription and higher rates of high-dose opioid prescription (Keyes et al 2014) compared to urban areas.

In the case studied here, factors such as the proportion of older people, the socioeconomic status, or access to health care services help to explain the opioid prescription rates and prescription pattern in La Gomera and its municipalities. Firstly, the prescription rate should increase in line with the rate of the rise in the population’s age. The results show that there is no significant correlation between the prescription opioid use rate, expressed as DID, and the population-aging rate at the municipal level. In order to evaluate the second factor, it is necessary to use an index of a social nature as measure of socioeconomic status similar to but necessarily the same as the Index of Social Deprivation described by Mordecai et al. (2018) used to evaluate socioeconomic status in the UK. This issue will be subject of future research.

Initially, practitioner needs to treat pain regardless of its etiology. For this, prescribing in primary care affects the nonsteroidal anti-inflammatory drugs and weak opioids used in treating chronic non-cancer pain in the short term. However, all medicine specialties (such as surgery, pain management services, oncology, sports medicine, etc.) that tend to be opioid prescribers are located at San Sebastian’s public hospital, the only one on the island, and thus, the prescription is initiated by a specialist. This can be an advantage with regard to indication for prescribing the opioid and with regard to dosage used. This could explain the opioid prescription rates and pattern observed in La Gomera. In this regard, two studies have demonstrated that the number of active clinicians or the number of general practitioners available in a geographic area could explain the opioid prescription rate (McDonald et al. 2012; NHS Digital 2021) . A similar prescription pattern was observed in the island of El Hierro, with half the number of municipalities and population compared to La Gomera, but with the same sociodemographic characteristics as well as access to specialist health services located in the capital, Valverde.

The use of health areas as a criterion does not provide any relevant or additional information to that obtained when referring to the municipalities for both indicators. This finding has also been observed on the islands of El Hierro and Fuerteventura in the Canary Islands (data not shown).

## Conclusions

This work shows that the information provided by the wholesalers who supply the community pharmacies is valuable, although limited since patients’ demographic data (age and gender) or medical data were not provided. Furthermore, the data provided no knowledge of indications, effects, or side effects related to the dispensed prescriptions. This is a limitation.

The overall picture of prescription opioid use was fairly stable at the island, municipal, and health area levels, although there are small variations. At municipal level, this variation over the year in prescription could be overestimated, although the potential bias is difficult to quantitate since the prescription is associated to pharmacy postal code (ZIP) and not township where the patient lives. This is a second limitation.

As regards the group of weak opioids, tramadol as monotherapy or in combination with acetaminophen and dexketoprofen are the most consumed weak opioids, always in presentations of low doses and low number of units per package. In the case of strong opioids, presentations with high doses and low number of units per presentations are more commonly prescribed, such as buprenorphine and fentanyl.

There are noteworthy changes in the balance between uses of weak or strong opioids in La Gomera in comparison with the rest of Spain. Prescription of weak opioids was 58.67% in La Gomera versus 68.28% in Spain as a whole, while for the prescription of strong opioids was higher than the national average (41.33% vs 31.72%). Factors such as the proportion of older people, socioeconomic status, and access to health care services or differences between rural–urban areas fail to explain the opioid prescription rates and prescription patterns in La Gomera and its municipalities. Additional research is needed to understand how the social and economic factors may affect the opioid prescribing practices described in this study. However, specialist physicians located in only one place (i.e., centralized services) initiate the prescription of opioids which is the characteristic of the islands of La Gomera and El Hierro. Therefore, this factor plays an important role in determining opioid prescribing rates.

## Data Availability

The data that support the findings of this study are available from COFARTE and COFARES, but restrictions apply to the availability of these data, which were used under license for the current study, and so are not publicly available. Data are however available from the corresponding author upon reasonable request and with permission of COFARTE and COFARES.
